# Supplementary material for: Variations in length of stay among surviving preterm infants admitted to neonatal intensive care units in Shenzhen, China
Source: PeerJ. 2025 Dec 8;13:e20344. doi: 10.7717/peerj.20344 (PMC12697294; doi:10.7717/peerj.20344)
Supplement: Supplemental Information 2 — Hospital length of stay and hospital costs were summarized descriptively by gestational age and weight. Quantitative baseline characteristics of the normal distribution are expressed as means with standard deviations, whereas highly skewed variables are expressed as medians with interquartile ranges. [file peerj-13-20344-s002.docx]

| **Table S1. Length of stay, corrected gestational age at discharge and hospitalization costs of preterm infants by gestational age and birth weight in neonatal intensive care units in Shenzhen, China.** | | | | |
| --- | --- | --- | --- | --- |
| **Characteristics** | **Number of infants** | **LOS (d), median (IQR)** | **cGA at discharge**  **(wk),median (IQR)** | **Costs（RMB）,median (IQR)** |
| **GA (wk)** |  |  |  |  |
| ≤24 | 7 | 110（103-117） | 40.3（39.6-41.6） | 257051.6(231139.8-310879.6) |
| 25 | 11 | 96（84-111） | 38.9（37.3-40.9） | 209046.8(175347.9-239148.2） |
| 26 | 19 | 79（70-85） | 37.9（36.6-38.4） | 158089.8（120063.9-182091.2） |
| 27 | 45 | 72（63.0-79.5） | 37.6(36.5-38.4) | 135020.2（122814.9-162849.8） |
| 28 | 52 | 63（53.3-71.8） | 37.4(36.3-38.3) | 120697.3（100420.8-145507.5） |
| 29 | 66 | 53.5（47-61） | 36.9(36.1-38.2) | 97458.2（79638.5-121212.6） |
| 30 | 92 | 44（39-53） | 36.6(36-37.9) | 84740.7（66487.4-103641.3） |
| 31 | 88 | 34（28-45.8） | 36.4(35.6-37.9) | 62871.9（44308.1-84056.7） |
| 32 | 157 | 28（23.5-34） | 36.4(35.9-37.1) | 49095.6（34865.5-60294.6） |
| 33 | 256 | 19（14-25） | 36.1(35.6-37) | 29148.2（21293.8-39799.8） |
| 34 | 436 | 13（10-16） | 36.3(35.9-36.7) | 19987.9（14487.9-26484.5） |
| 35 | 683 | 9（7-11） | 36.7(36.4-37.1) | 13929.2（9618.2-18041.2） |
| 36 | 975 | 7（5-8） | 37.4(37.1-37.7) | 10806.3（6529.4-14212.1） |
| **Birth weight (kg)** |  |  |  |  |
| <1000 | 91 | 77（66-88） | 38.4（37.1-40） | 148761.3（120063.9-189979.5） |
| 1000-1249 | 115 | 57（49-66） | 37.9（36.6-38.7） | 101449.3（80792.1-132280.33） |
| 1250-1499 | 150 | 43（33-51） | 36.9（36.1-38.0） | 80382.8(53135.1-99383.1) |
| 1500-2499 | 1472 | 12（8-20） | 36.7（36.1-37.4） | 18675.2(12975.5-30238.3) |
| ≥2500 | 1059 | 7（5-9） | 37.1（36.6-37.4） | 11275.3(6943.8-15215.8) |
| LOS, length of stay; IQR, interquartile range; GA, gestational age; cGA, corrected gestational age | | | | |
