# Supplementary material for: Variations in length of stay among surviving preterm infants admitted to neonatal intensive care units in Shenzhen, China
Source: PeerJ. 2025 Dec 8;13:e20344. doi: 10.7717/peerj.20344 (PMC12697294; doi:10.7717/peerj.20344)
Supplement: Supplemental Information 3 — Generalised linear models (GLMs) with gamma distribution were used to determine the association between perinatal factors and neonatal complications with hospitalisation costs. [file peerj-13-20344-s003.docx]

| **Table S2. Factors affecting the hospitalization costs for surviving preterm infants in neonatal intensive care units in Shenzhen, China.** | | | | |
| --- | --- | --- | --- | --- |
| **Factors** | Model 1 | Model 2 | Model 3 | Model 4 |
|  | RR(95%CI) | RR(95%CI) | RR(95%CI) | RR(95%CI) |
| **Maternal characteristics** |  |  |  |  |
| Maternal age | 1.001(0.99, 1.00) |  |  |  |
| Primigravida | 1.04(0.98, 1.12) |  |  |  |
| Maternal hypertension | **1.41(1.29, 1.54)** | **1.13(1.08, 1.19)** |  | **1.13(1.08, 1.19)** |
| Maternal diabetes | 1.04(0.96, 1.12) |  |  |  |
| GBS infection | 0.88(0.75, 1.03) |  |  |  |
| Antenatal corticosteroids | **1.82(1.70, 1.94)** | **1.04(1.001, 1.08)** |  | **1.05(1.01, 1.09)** |
| cesarean section | 1.05(0.97, 1.13) |  |  |  |
| PROM ≥ 18 hours | **1.31(1.20, 1.44)** | **1.07(1.02, 1.13)** |  | **1.07(1.01, 1.13)** |
| **Infant characteristics** |  |  |  |  |
| GA(wk) |  |  |  |  |
| <28 | Reference | Reference |  | Reference |
| 28-31^+6^ | **0.55(0.48,0.62)** | **0.83(0.72, 0.97)** |  | **0.86(0.73, 1.01)** |
| 32-33^+6^ | **0.24(0.21, 0.27)** | **0.61(051,0.72)** |  | **0.63(0.52, 0.76)** |
| 34-36^+6^ | **0.09(0.08, 0.10)** | **0.32(0.27,0.38)** |  | **0.33(0.27, 0.39)** |
| Birth weight (g) |  |  |  |  |
| <1000 | Reference | Reference |  | Reference |
| 1000-1249 | **0.68(0.58, 0.80)** | 0.95(0.82, 1.10) |  | 1.08(0.93, 1.25) |
| 1250-1499 | **0.49(0.42, 0.57)** | **0.82(0.70, 0.96)** |  | 0.96(0.82, 1.13) |
| 1500-2499 | **0.15(0.14, 0.17)** | **0.58(0.50, 0.69)** |  | **0.72(0.61, 0.85)** |
| ≥ 2500 | **0.08(0.07, 0.09)** | **0.44(0.37, 0.52)** |  | **0.57(0.48, 0.69)** |
| SGA | **1.28(1.15, 1.43)** | **1.11(1.04, 1.18)** |  | 1.02(0.95, 1.09) |
| Male | 0.97(0.90, 1.03) |  |  |  |
| Multiple birth | **1.08(1.001, 1.16)** | **0.96(0.92, 0.99)** |  | **0.96(0.92, 0.99)** |
| Apgar score ≤ 7 at 1 min | **3.4(2.97, 3.90)** | **1.17(1.07, 1.27)** |  | **1.15(1.06, 1.25)** |
| Apgar score ≤ 7 at 5 min | **3.71(2.73, 5.04)** | 1.15(0.97, 1.37) |  | 1.16(0.97, 1.39) |
| surfactant use | **4.67(4.33, 5.03)** | **1.84(1.73, 1.95)** |  | **1.82(1.71, 1.93)** |
| **Major infant morbidities** |  |  |  |  |
| nosocomial infection | **2.71(2.43, 3.02)** |  | **1.50(1.35, 1.66)** | 1.05(0.99, 1.12) |
| IVH grade III and above or cPVL | **3.03(2.18, 4.21)** |  | **1.71(1.29, 2.27)** | 1.07(0.90, 1.29) |
| NEC ≥ stage II | **3.08(2.05, 4.64)** |  | **2.2(1.56, 3.12)** | **1.36(1.09, 1,69)** |
| BPD | **5.87(5.09, 6.78)** |  | **4.35(3.78, 5.02)** | **1.13(1.001, 1.28)** |
| Severe ROP | **6.09(3.92, 9.46)** |  | **1.58(1.06, 2.36)** | **1.31(1.01, 1.69)** |
| Sepsis | **3.64(2.97, 4.46)** |  | **2.05(1.71, 2.46)** | **1.29(1.15, 1.45)** |
| EUGR | **1.45 (1.35, 1.57)** |  | **1.34(1.26, 1.43)** | **1.18 (1.12, 1.24)** |

GBS, Group B Streptococcus; PROM, premature rupture of membranes; LOS, length of stay; GA, gestational age; SGA, small for gestational age; IVH ,intraventricular hemorrhage; cPVL, cystic periventricular leukomalacia; NEC, necrotizing enterocolitis; BPD, bronchopulmonary dysplasia; ROP, retinopathy of prematurity; EUGR, extrauterine growth restriction. RR:Relative Risk. Model 1：Crude; Model 2：Adjust: maternal hypertension, antenatal corticosteroids, gestational age,Birth weight ,small for gestational age, multiple birth, surfactant use,Apgar score ≤ 7 at 1 min,Apgar score ≤ 7 at 5 min and preterm rupture of membranes ≥ 18 hours; Model 3：Adjust: nosocomial infections ,IVH grade III and above or cPVL, NEC ≥ stage II, BPD, severe ROP ,sepsis and EUGR. Model 4：Adjust Model 2 plus Model 3. Significant results are in boid.
